# Transcriptional repression of FOXO1 by KLF4 contributes to glioma progression

**DOI:** 10.18632/oncotarget.13184

**Published:** 2016-11-07

**Authors:** Guodong Tang, Dingyang Liu, Gelei Xiao, Qing Liu, Jian Yuan

**Affiliations:** ^1^ Department of Neurosurgery, Xiangya Hospital, Central South University, Changsha 410008, Hunan, China; ^2^ The Institute of Skull Base Surgery and Neurooncology at Hunan Province, Changsha 410008, Hunan, China

**Keywords:** glioma, FOXO1, progression, KLF4, transcriptional regulation

## Abstract

In this study, our findings indicated that FOXO1 expression frequently decreased in glioma tissues and cells. FOXO1 expression decrease correlated with glioma progression and predicted a worse overall survival of glioma patients. Restored FOXO1 expression inhibited glioma cells invasion and suppressed glioma cells proliferation in vitro and growth in vivo. Additionally, we found that KLF4 expression frequently increased in glioma tissues and negatively correlated with FOXO1 expression. Bioinformatics analysis and experimental results indicated that KLF4 transcriptionally repressed FOXO1 expression in glioma cells. Moreover, KLF4 expression increase correlated with glioma progression and predicted a poorer overall survival of glioma patients. KLF4 knockdown attenuated glioma cells invasion and growth. These data provide a rationale for targeted intervention on KLF4-FOXO1 signaling pathway to suppress glioma progression.

## INTRODUCTION

Malignant glioma, is the most common and type of malignant primary brain tumors in human and is characterized by high morbidity and mortality rates. Malignant glioma is typically aggressive, highly infiltrative and resistant to conventional therapy [1]. Despite the advancements in both diagnostic modalities and therapeutic strategies over the past several decades, the prognosis of malignant glioma still remains poor [2]. This poor prognosis is mainly due to the invasive potential of malignant glioma, which precludes complete resection and enhances resistance to therapy [3]. Thus, currently it is urgent to elucidate the biology and molecular mechanisms of glioma development and progression, based on which to develop more effective treatments

FOXO1 is a transcription factor and a member of the FOXO family that has four members: FOXO1, FOXO3, FOXO4, and FOXO6 in mammalian [4]. Recently, FOXOs have been widely studied for their broad roles in physiological process, including cell cycle arrest, apoptosis, angiogenesis, stress resistance, energy metabolism, and stem cell differentiation [5]. The FOXO1 protein level and transcriptional activation are tightly regulated by multiple posttranslational modifications, including phosphorylation, acetylation, ubiquitination and methylation [6]. Previous studies have suggested that FOXO1 functions as a tumor suppressor. Decreased FOXO1 expression has been shown in many cancer types, such as Hodgkin lymphoma [7], breast cancer [8] and alveolar rhabdomyosarcoma [9]. Furthermore, FOXO1 suppressed the metastatic potential through inhibiting MMP7 in larynx cancer cells [10] or MMP9 in lung cancer cells [11]. FOXO1 inhibits angiogenesis in gastric cancer via inactivation of the HIF-1α-VEGF pathway [12] and inhibits cell growth, tumorigenesis and chemo-resistance in nasopharyngeal carcinoma [13]. However, the precise expression pattern and role of FOXO1 in glioma remain elusive.

Here, the objective of the present study was to investigate the expression pattern, function, clinical significance and regulatory mechanism of FOXO1 in glioma. Our findings indicated that FOXO1 down-regulation mediated by KLF4 confers to progression of glioma.

## RESULTS

### FOXO1 expression is repressed in glioma

**Figure 1:**
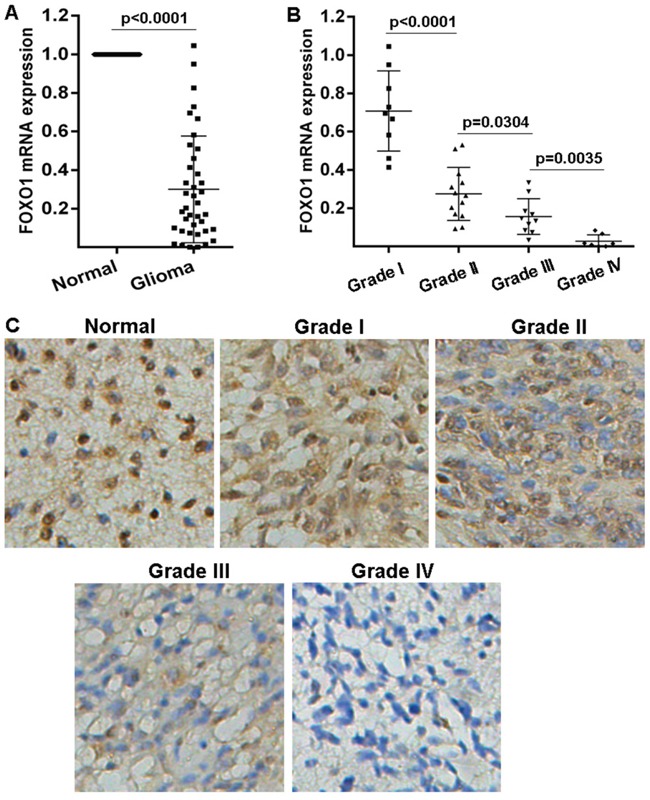
FOXO1 expression pattern in glioma **A.** FOXO1 mRNA level in glioma tissue samples represented as fold change were detected with qRT-PCR by normalizing to GAPDH as endogenous control and the expression level in matched non-tumor tissues was set as 1. **B.** The correlation between FOXO1 mRNA expression and glioma grades was analyzed. **C.** Representative images of FOXO1 protein level in glioma tissue samples detected by IHC (20×).

In order to investigate the role of FOXO1 in glioma, here the FOXO1 mRNA level in the 39 fresh-frozen human primary glioma tissue samples with different grades was examined using qRT-PCR. Results indicated that FOXO1 mRNA level significantly decreased in the majority of primary glioma tissue samples, compared with the matched non-tumor brain tissues (Figure 1A). Additionally, the decrease of FOXO1 mRNA level in the high-grade gliomas was greater than that in the low-grade gliomas (Figure 1B). Then, the FOXO1 protein level in a cohort of 73 tissue samples involving 8 normal brain tissue samples and 65 glioma tissue samples with follow-up records, was examined using immunohitochemical staining,. Compared with nor-tumor brain tissue samples, FOXO1 protein level was significantly down-regulated in the majority of primary glioma tissue samples, with a greater decrease in high-grade gliomas than in low-grade (Figure 1C). These results indicated that FOXO1 expression is repressed in gliomas.

### FOXO1 down-regulation is associated with glioma progression

**Figure 2:**
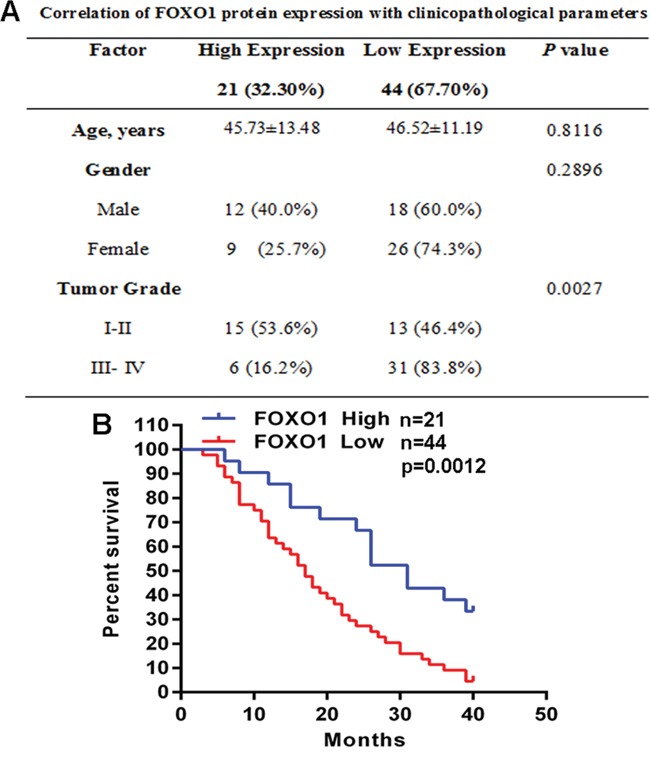
FOXO1 down-regulation is associated with glioma progression **A.** The correlation analyses of FOXO1 protein level in relation to clinicopathologic variables of glioma patients. **B.** Kaplan-Meier analysis estimated the overall survival according to FOXO1 protein level.

To investigate the role of FOXO1 in glioma, we analyzed the correlation of FOXO1 protein level with clinicopathological parameters in the cohort of 65 glioma tissue samples. Notably, the low FOXO1 protein level was associated with higher tumor grade (Figure 2A). Furthermore, for the analysis of overall survival (OS), glioma patients group with low FOXO1 protein level had significantly poorer OS than the patients group with high FOXO1 protein level (Figure 2B). These analyses demonstrated that FOXO1 is associated with glioma progression and would be a valuable predictor for the survival of glioma patients.

### FOXO1 inhibits glioma cells invasion and growth

**Figure 3:**
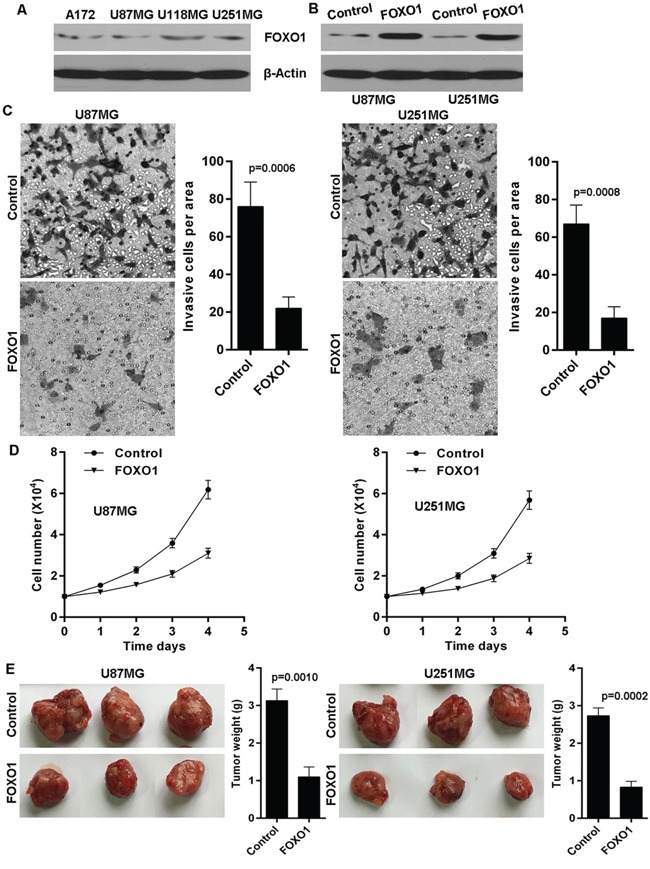
FOXO1 suppresses glioma cells invasion and growth **A.** Western blot analysis of FOXO1 protein level in glioma cell lines. **B.** FOXO1 was stably overexpressed in U87 and U251 cell lines confirmed by western blot. **C.** FOXO1 overexpression decreased invasion potential of U87 and U251 cell lines, as shown by cell counts per area. **D.** FOXO1 overexpression inhibited proliferation potential of U87 and U251 cell lines in vitro determined cell number count. **E.** Tumor size and weight were recorded. Data are represented as a mean ± SD from three mice.

The above findings imply the involvement of FOXO1 in human glioma. Thus, we further performed experiments to explore the biological functions of FOXO1 in glioma cells. We firstly detected FOXO1 protein level in a series of human glioma cell lines and found that FOXO1 protein was relatively lowly expressed in the A172, U87MG, U118MG and U251MG glioma cell lines, compared with endogenous control β-Actin (Figure 3A). Here, we then established stable FOXO1 overexpression glioma cell lines in U87MG and U251MG via transfection with FOXO1 overexpressing plasmid pReceiver-Lv/FOXO1 tested by western blot (Figure 3B). The effect of FOXO1 on cell invasion in glioma cells was determined using transwell assay. As shown, FOXO1 overexpression significantly inhibited the invasive potential of U87MG and U251MG cells (Figure 3C). Moreover, ectopic FOXO1 expression markedly inhibited U87MG and U251MG cells proliferation in vitro (Figure 3D). We further confirmed the effect of FOXO1 on proliferation of glioma cells in vivo using nude mice model with glioma xenografts. We injected subcutaneously into the oxter of athymic mice with FOXO1 overexpressing U87MG and U251MG cell lines and their related control cell lines. After 4 weeks, the mice bearing tumors were euthanized, the tumors were excised and the wet weights of the tumors were recorded. Tumors induced by FOXO1 overexpression cells were significantly smaller and lighter than tumors induced by control cells (Figure 3E).

### KLF4 expression inversely correlated with FOXO1 expression

**Figure 4:**
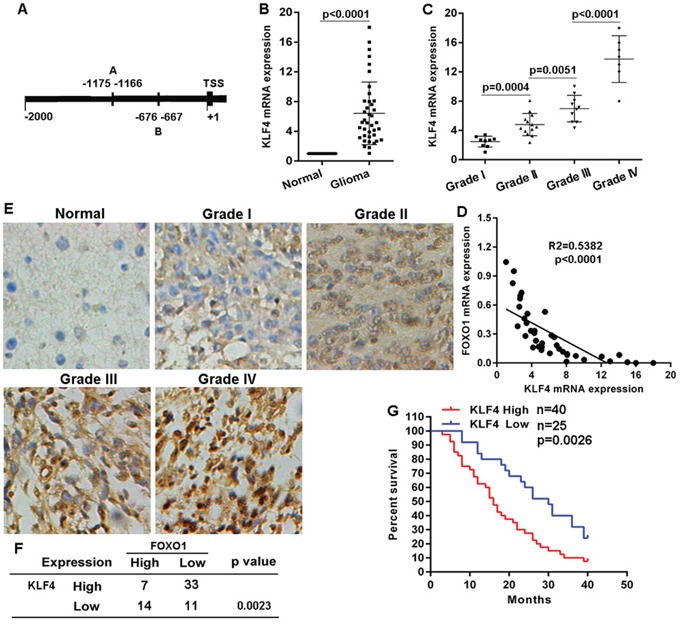
KLF4 negatively correlated with FOXO1 expression in glioma **A.** Schematic of the putative binding site of KLF4 in the FOXO1 promoter. **B.** KLF4 mRNA level in glioma tissue samples represented as fold change were detected with qRT-PCR by normalizing to GAPDH as endogenous control and the expression level in matched non-tumor tissues was set as 1. **C.** The correlation between KLF4 mRNA expression and glioma grades was analyzed. **D.** Pearson's correlation analyses between relative KLF4 mRNA and FOXO1 mRNA levels in glioma tissues. **E.** Representative images of KLF4 protein level in glioma tissue samples detected by IHC (20×). **F.** The negative correlation between KLF4 protein and FOXO1 protein level in glioma tissues was analyzed. **G.** Kaplan-Meier curves showing the overall survival of patients with high or low protein level of KLF4 in their gliomas. Statistical significance was determined by a log-rank test.

To observe transcriptional regulation of FOXO1 expression in glioma, we analyzed the response elements of a cohort of transcription factors located within the two kilobase region upstream of transcription start site of *FOXO1* gene. Using the JASPAR database (http://jaspar.binf.ku.dk) we identified two putative KLF4 binding sites within this region (Figure 4A), suggesting KLF4 overexpression resulting into FOXO1 repression. KLF4 mRNA level in the 39 fresh-frozen human primary glioma tissue samples with diverse grades was detected using qRT-PCR. KLF4 mRNA level significantly increased in the majority of primary glioma tissue samples, compared with matched non-tumor brain tissues (Figure 4B). Additionally, the increase of KLF4 mRNA level in high-grade gliomas was greater than in low-grade gliomas (Figure 4C). Expectedly, KLF4 mRNA level was inversely correlated with FOXO1 mRNA level in glioma tissue samples (Figure 4D). Then, we also examined KLF4 protein level in the cohort of 73 tissue samples using immunohitochemical staining. Compared with normal brain tissue samples, KLF4 protein level was significantly up-regulated in the majority of primary glioma tissue samples, with a greater increase in high-grade gliomas than in low-grade (Figure 4E). KLF4 protein level was also inversely correlated with FOXO1 protein level in the glioma tissue samples (Figure 4F). Furthermore, for the analysis of overall survival (OS), glioma patients group with high KLF4 protein level had significantly worse OS than the patients group with low KLF4 protein level (Figure 4G). These investigations indicated a negative correlation between KLF4 and FOXO1 in gliomas.

### KLF4 transcriptionally inhibits FOXO1 expression in glioma cells

**Figure 5:**
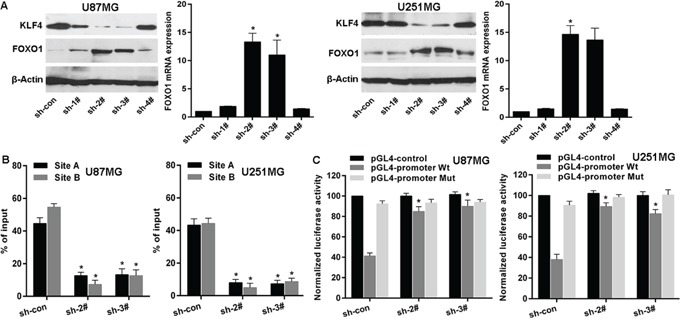
KLF4 transcriptionally represses FOXO1 expression in glioma **A.** FOXO1 expression in mRNA and protein levels was detected by qRT-PCR and western blot after KLF4 knockdown. *vs* control, * *p*<0.0001. **B.** ChIP-qPCR for the KLF4 binding to the FOXO1 promoter in KLF4 knockdowned cell lines by transfection of shRNAs. *vs* control, * *p*<0.001. **C.** Luciferase reporter assay for the luciferase activity driven by FOXO1 promoter in U87 or U251 cell lines after KLF4 knockdown. *vs* control, * *p* <0.001.

To examine the role of KLF4 in regulating FOXO1 expression, we firstly used shRNAs to knockdown KLF4 expression in U87MG and U251MG cells (Figure 5A). Next, qRT-PCR analysis and western blot indicated that FOXO1 expression was markedly increased in cell lines after KLF4 knockdown (Figure 5A), suggesting that KLF4 is an upstream regulator of FOXO1. KLF4 was stably knockdowned in U87MG and U251MG cell lines using sh-2# and sh-3#. Then, Chromatin immunoprecipitation-qPCR (ChIP-qPCR) assay further confirmed that KLF4 protein was exactly recruited to the two binding sites in the putative FOXO1 promoter in U87MG and U251MG cell lines and that KLF4 knockdown resulted into decrease of the binding level (Figure 5B). Furthermore, to confirm the effect of KLF4 on the transcriptional activation of *FOXO1* promoter, the *FOXO1* promoter was cloned into the pGL4 reporter plasmid. Experimental results indicated an increase of the wild-type *FOXO1* promoter luciferase activity was observed in U87MG and U251MG cell lines after KLF4 knockdown, but without significant change on the binding sites mutant-type (Figure 5C). These data suggest that KLF4 binds to *FOXO1* promoter and inhibits its transcription in glioma cells.

### KLF4 knockdown suppresses glioma cells invasion and growth

**Figure 6:**
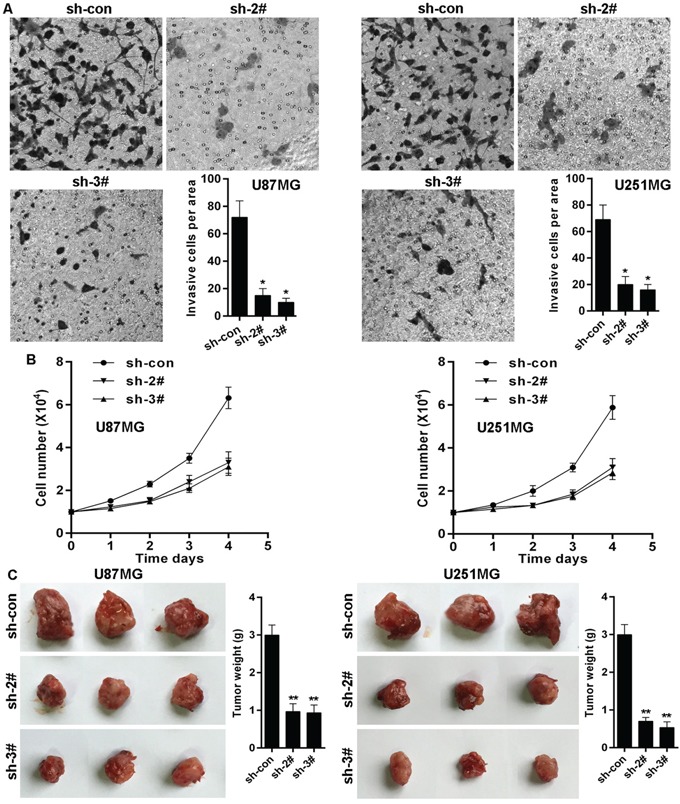
KLF4 knockdown suppresses glioma cells invasion and growth **A.** KLF4 knockdown decreased invasion potential of U87 and U251 cell lines, as shown by cell counts per area. **B.** KLF4 knockdown inhibited proliferation potential of U87 and U251 cell lines in vitro determined cell number count. **C.** KLF4 knockdown inhibited U87 and U251 cells growth in vivo. Data are represented as a mean ± SD from three mice. vs control, * *p*<0.001.

Given that FOXO1 inhibits glioma cells invasion and growth, and that KLF4 transcriptionally inhibits FOXO1 expression, we here also observed the effects of KLF4 on invasion and growth in glioma cells. The effect of KLF4 on cell invasion in glioma cells was determined using transwell assay. As shown, KLF4 knockdown significantly inhibited the invasive potential of U87MG and U251MG cells (Figure 6A). Moreover, KLF4 knockdown markedly inhibited U87MG and U251MG cells proliferation in vitro (Figure 6B). We further confirmed the role of KLF4 in growth of glioma cells in vivo using nude mice with glioma xenografts. We injected subcutaneously into the oxter of athymic mice with KLF4 knockdowned U87MG and U251MG cell lines and their related control cell lines. After 4 weeks, the mice bearing tumors were euthanized, the tumors were excised and the wet weights of the tumors were recorded. Tumors induced by KLF4 knockdowned cells were significantly smaller and lighter than tumors induced by control cells (Figure 6C).

## DISCUSSION

Although the tumor suppressive role of FOXO1 has been well characterized for some cancer types, little is known of its biological function and significance in glioma. In the present study, we demonstrated the expression pattern, function and clinical significance of FOXO1 in glioma. Our data indicated that FOXO1 expression is down-regulated in glioma tissue samples and cells, and acts as a cancer suppressor. We found that FOXO1 is frequently down-regulated at both mRNA and protein levels in glioma, and that FOXO1 down-regulation was correlated with glioma progression. Invasion and fast growth are usually associated with the poor outcome of glioma patients. Our results showed that ectopic FOXO1 expression inhibited glioma cells invasive potential and inhibited glioma cells growth in vitro and in vivo. Thus, our data provide direct evidence that FOXO1 acts as a cancer suppressor in glioma cells via modulating oncogenic behaviors. Recently, FOXO1 was shown to inhibit osteosarcoma oncogenesis via wnt/β-catenin pathway suppression [14]. The precise mechanism underlying the cancer-suppressive properties of FOXO1 in glioma remains elusive in our further studies.

The FOXO1 protein level and transcriptional activation are tightly regulated by multiple posttranslational modifications, including phosphorylation, acetylation, ubiquitination and methylation [15]. The phosphorylation of FOXO1 by AKT leads to its inactivation resulted from nuclear to cytoplasmic translocation [16, 17]. TXN curtails p300-mediated FOXO1 acetylation and its nuclear translocation in response to oxidative stress, thus attenuates FOXO1 transcriptional activity toward genes involved in apoptosis and cell cycle inhibition in diffuse large B-cell lymphomas [18]. Here, we found that FOXO1 is frequently down-regulated at both mRNA and protein levels in glioma, suggesting FOXO1 expression should be regulated at transcriptional level. To sought and identify transcription factors conferring *FOXO1* down-regulation in glioma cells, bioinformatics analysis and experimental data indicated that KLF4 physically binds to the *FOXO1* promoter and inhibits the activity of *FOXO1* promoter. KLF4 knockdown up-regulated *FOXO1* expression at both mRNA and protein levels in glioma cells. Importantly, the negative association between KLF4 and FOXO1 levels was observed in glioma tissue samples. KLF4 was initially identified in postmitotic, terminally differentiated epithelial cells of the skin and the gastrointestinal tract [19, 20]. KLF4 is a zinc finger transcription factor that is involved in regulating numerous physiological processes, such as proliferation, development, apoptosis, differentiation, maintenance of tissue homeostasis and carcinogenesis [21]. It is well known that KLF4 is a core component of the pluripotency transcription network that has been widely used to reprogram somatic cells into induced pluripotent stem cells [22, 23]. Additionally, KLF4 exhibits anti-apoptotic activity upon DNA damage via modulating the functions of p53 and via blocking cell cycle progression at the G1/S boundary [24, 25]. There is mounting evidence indicating that KLF4 functions as a tumor suppressor or tumor promoter, depending on the cellular context. KLF4 has been reported to act as a tumor suppressor in some cancer types, such as lung carcinoma [26], gastric cancer [27] and colorectal cancer [28]. Conversely, an oncogenic function of KLF4 has also been reported in primary breast ductal carcinoma and squamous carcinoma of oral and skin. In primary breast ductal carcinoma, KLF4 maintains the stem cell-like features to enhance invasion and migration [29]. KLF4 also plays crucial roles in maintenance of neural stem cells, and KLF4 deficiency causes impaired neurogenesis in adult mouse brain [30, 31]. KLF4 induced by PGI initiated by mesenchymal glioma cells, induces the self-renewal and tumorigenic potentials of glioma stem cells [32]. These reports and our findings suggest the potential role of KLF4 in glioma.

In summary, here we investigated the expression pattern and role of FOXO1 in glioma. Our findings indicated that FOXO1 is frequently down-regulated in glioma and its down-regulation is correlated with glioma progression. Restored FOXO1 expression inhibits glioma cells growth and invasion. KLF4 transcriptionally represses FOXO1 expression. Thus, KLF4-FOXO1 signaling could be a useful biomarker for predicting the progression of glioma and may provide new clue to develop effective therapeutic strategies.

## MATERIALS AND METHODS

### Clinical specimens, cell culture and transfection

The 39 paired fresh primary human glioma tissue samples and adjacent non-tumor tissues, and the 73 formalin-fixed paraffin-embedded tissue samples were obtained from the Department of Neurosurgery at Xiangya Hospital of Central South University. Fresh tissue samples after resection were immediately snap-frozen in liquid nitrogen used for subsequent RNA extraction. Ethical approval for human subjects was got from the research and ethics committee of the Xiangya Hospital of Central South University and informed consent was obtained from each patient. Human glioma cell lines A172, U87MG, U118MG and U251MG were obtained from and maintained as recommended by the American Type Culture Collection (ATCC, Manassas, VA, USA). Cells were cultured in DMEM medium supplemented with 10% fetal bovine serum (Gibco, Carlsbad, CA, USA). For FOXO1 overexpression and KLF4 knockdown, cells were transfected with FOXO1 overexpressing plasmid pReceiver-Lv/FOXO1 and pSUPER plasmids containing KLF4 specific shRNAs respectively using Lipofectamine 2000 (Invitrogen, Carlsbad, CA, USA) in accordance with the manufacturer's procedure.

### Real-time PCR (qRT-PCR) for mRNA

Total RNA from tissue samples and cell lines was extracted with a Trizol protocol. The cDNAs from mRNAs were synthesized using the Super-Script first-strand synthesis system (Thermo Scientific, Glen Brunie, MA, USA). Real-time PCR was performed according to the standard protocol on ABI 7500 with SYBR Green detection (Applied Biosystems, Foster City, CA, USA). GAPDH was used as an internal (no differential expression) control. The qRT-PCR was repeated biologically three times. The primers for GAPDH were: forward primer 5'-AGGTCGGTGTGAACGGATTTG-3', reverse primer 5'-TGTAGACCATGTAGTTGAGGTCA-3'; for FOXO1 were: forward primer 5'-CCCAGGCCG GAGTTTAACC-3', reverse primer 5'-GTTGCTCATAA AGTCGGTGCT-3'; for KLF4 were: forward primer 5'-GTGCCCCGACTAACCGTTG-3', reverse primer 5'-GTCGTTGAACTCCTCGGTCT-3'.

### Immunohistochemistry assay

Immunohistochemistry (IHC) staining was performed as described in our previous study [33]. Briefly: Formalin-fixed, paraffin-embedded tissue specimens were cut into 4-μm sections. The specimens were deparaffinized in xylene and rehydrated using a series of graded alcohols after being dried at 62°C for 2 hrs. The tissue slides were then treated with 3% hydrogen peroxide in methanol for 15 min. To exhaust endogenous peroxidase activity, and the antigen were retrieved in 0.01 M sodium cirate buffer (pH 6.0) using a microwave oven. After 1 h of preincubation in 10% goat serum, the specimens were incubated with primary antibody overnight at 4°C. The tissue slides were treated with a non-biotin horseradish peroxidase detection system according to the manufacturer's instruction (DAKO, Glostrup, Denmark). Two different pathologists who specialize in gliomas evaluated the results of IHC.

### Western blot

Western blot was performed as described in our previous study [33]. Total proteins were extracted from corresponding cells, loaded and separated on 10% SDS-PAGE, and then transferred to PVDF membrane (Millipore, Billerica, MA, USA). The primary antibodies used for analysis included rabbit anti-FOXO1 monoclonal antibody, rabbit anti-KLF4 monoclonal antibody and mouse anti-β-Actin monoclonal antibody were from Cell Signaling Technology (Danvers, MA, USA).

### Transwell assay

Transwell assay was performed as described in our previous study. Briefly: Cells were detached and resuspended in serum-free medium. Cells (1 × 10^5^ cells/well) were then plated into Matrigel coated invasion chambers (Becton Dickinson) and allowed to invade for 24 hours. The remaining cells in the chambers were removed by cotton swabs and the invading cells on the lower surface of the chambers were fixed with 70% ethanol and then stained with hematoxylin. The number of invading cells was calculated by counting three different fields under a phasecontrast microscope.

### Xenograft model in nude mice

Xenograft tumours were generated by subcutaneous injection of FOXO1 overexpressed U87MG and U251MG cell lines, KLF4 knockdowned U87MG and U251 cell lines, or related control cell lines (2 × 10^6^), into the oxter of 4-6 week-old Balb/C athymic nude mice. All mice were housed and maintained under specific pathogen-free conditions, and all experiments were approved by the Use Committee for Animal Care and performed in accordance with institutional guidelines. After 4 weeks, the mice bearing tumors were euthanized, the tumors were excised and the wet weights of the tumors were recorded.

### ChIP-qPCR

The ChIP assay was performed using the EZ-CHIP™ chromatin immunoprecipitation kit (Merck Millipore) as previously described [34]. Briefly: before formaldehyde crosslinking, cells were treated with 10 mM dimethyl adipimidate (DMA) 0.25% DMSO in PBS for 45 min. Chromatin was then sheared to an average length of 0.25–1 kb. KLF4 antibody was used to immunoprecipitated with the aforementioned DNA. Primers for the *FOXO1* promoter in ChIP-qPCR assay were: for Site A, sense: 5'-CTTCTGCTTGAGACACAAGGG-3', antisense: 5'-CGGGAGATAGGACCAAAGCCTTGG-3'; for Site B, sense: 5'-CTGCCGGCTGGGTGACGCG-3', antisense: 5'-TCCTGGCTCCACCCACGATG-3'. qRT-PCR was carried out according to the standard protocol on ABI 7500 with SYBR Green detection (Applied Biosystems). The results were calculated by normalizing to the positive control, and relative quantization values were calculated using % positive control = 2^(-ΔCt [(Ct [FOXO1] - (Ct [positive control]]) method.

### Promoter activity analysis

To determine whether KLF4 regulates the promoter activity of FOXO1, a two kilobase region upstream of the transcription start site of *FOXO1* was cloned into the pGL4-reporter vector upstream of the luciferase gene. The promoter activity was detected as previously described [34]. Cells were seeded in 96-well plates and co-transfected with the pGL4-reporter vector and the pRL-TK Renilla luciferase vector with or without the pSUPER-sh-KLF4 vector using Lipofectamine 2000 (Invitrogen). After 48 h, luciferase activity was determined using a Dual-Luciferase Reporter Assay System (Promega) on the BioTek Synergy 2. Renilla luciferase activity was used as an internal control and the firefly luciferase activity was calculated as the mean ±SD after normalization relative to the Renilla luciferase activity.

### Statistical analysis

All statistical analyses were performed with SPSS statistical software (version 21.0; IBM). Survival curves were constructed using the Kaplan–Meier method and analyzed by the log-rank test. The Student's t-test was used for comparisons and the Pearson correlation test (two-tailed) was used to investigate the correlation. Statistical significance was defined as *p* < 0.05.
